# The use of adjuvant chemotherapy in cats with mammary carcinomas undergoing surgical removal

**DOI:** 10.18849/ve.v9i4.688

**Published:** 2024-11-18

**Authors:** Gabriela Gonzalez-Ormerod

**Keywords:** cat, chemotherapy, feline, mammary carcinoma, mastectomy, survival time

## Abstract

**PICO question:**

In cats with mammary carcinomas undergoing surgical removal, does the addition of adjuvant chemotherapy compared with surgical removal alone result in increased survival time?

**Clinical bottom line:**

**Category of research:**

Treatment.

**Number and type of study designs reviewed:**

Five retrospective cohort studies were critically reviewed.

**Strength of evidence:**

Weak.

**Outcomes reported:**

In cats with mammary carcinomas undergoing surgical removal, the addition of adjuvant chemotherapy compared with surgical removal alone was significantly associated with an increase in disease-specific survival time in one of the studies. This statistical significance was not found in the other four papers.

**Conclusion:**

All five studies reviewed presented weak evidence for the clinical question due to their retrospective nature and weak study design. It is, therefore, concluded that there is not enough evidence to suggest that cats with mammary carcinomas that have undergone surgical treatment will have a longer survival time if treated with adjuvant chemotherapy. More prospective, placebo-controlled, double-blinded studies are needed to understand the clinical significance of adjuvant chemotherapy in cats with mammary carcinomas.

## How to apply this evidence in practice

The application of evidence into practice should take into account multiple factors, not limited to: individual clinical expertise, patient’s circumstances and owners’ values, country, location or clinic where you work, the individual case in front of you, the availability of therapies and resources.

Knowledge Summaries are a resource to help reinforce or inform decision-making. They do not override the responsibility or judgement of the practitioner to do what is best for the animal in their care.

## Clinical scenario

A 10-year-old female neutered Domestic Shorthair cat “Candy” is brought to your clinic because her owners noticed a mass on her abdomen. The owners report that she is otherwise healthy and is eating, drinking, urinating and defecating normally. On physical examination you palpate a well-defined 4 cm mildly ulcerated mass associated with Candy’s right inguinal mammary gland. The rest of her mammary glands are normal on palpation. You take fine-needle aspirates of the mass and perform full bloods (haematology and biochemistry), urine analysis, abdominal ultrasonography and thoracic radiographs. Your workup confirms that Candy has a mammary adenocarcinoma and there is cytological evidence of spread to her inguinal lymph nodes without any evidence of distant metastasis. Candy has a stage III mammary carcinoma according to the modified World Health Organization (WHO) staging system. Her owners consent to a staged bilateral mastectomy but you have had a difficult conversation with them as stage III–IV mammary tumours are associated with a poorer prognosis and survival times despite surgery. Candy’s owners want to do absolutely everything to help prolong her life expectancy and have asked if adjuvant chemotherapy is an option. Is there any evidence to suggest that surgical treatment of mammary carcinomas plus adjuvant chemotherapy results in increased survival times?

## The evidence

The literature search revealed five retrospective cohort studies relevant to the PICO question (De Campos et al. (2014); Gemignani et al. (2018); Ito et al. (1996); McNeill et al. (2009); Petrucci et al. (2021)). All five studies presented weak evidence for the clinical question largely due to the fact that their retrospective nature meant that there was variability in patient’s disease stage, a lack of standardisation of surgical and chemotherapeutic protocols as well as the absence of placebo groups. There were also noticeable limitations when retrospectively determining disease-specific causes of death and survival times. Furthermore, there is limited applicability of the results in first opinion practices given that most of the data was obtained from referral teaching hospitals and/or specialised private practices outside of the UK.

## Summary of the evidence

**De Campos et al. (2014):** 

Population	Cats with mammary carcinomas admitted to the veterinary teaching hospital Federal University of Minas Gerais, Brazil.
Sample Size	16 cats.
Intervention Details	Group 1 (n = 9): cats treated with unilateral radical mastectomy. Group 2 (n = 7): cats treated with conventional surgical excision and four intravenous cycles of carboplatin (200 mg/m^2^) every 21 days.
Study design	Retrospective cohort study.
Outcome studied	Overall survival (days between the date of surgical removal of the tumour and death caused by the disease). Median survival (when 50% of cats from a determined group died).
Main findings (relevant to PICO question)	Median survival: Group 1: 387 days Group 2: 428 days. Overall survival time is not included in this paper. The differences in median survival times found between groups were not statistically significant (P = 0.883).
Limitations	Retrospective design weak for clinical question. No placebo group. No power calculations were performed. Limited information on population (other comorbidities, current medications, etc.). Population of cats in Brazil may not be representative of population of cats in the UK. Study performed in a referral level hospital and therefore applicability to first opinion may be limited. Treatment options were not randomised. The decision to start on adjuvant chemotherapy was left to owners. Not clear if both groups received the same type of surgical treatment. Accurately determining cause of death is complicated given that patient follow-up was based on owner phone calls and medical records. Median patient follow-up time was also low (202 days). No information was available on how long after surgery the cats were started on chemotherapy. Multivariate statistical analysis not performed.

Table note...

**Gemignani et al. (2018):** 

Population	Cats that underwent unilateral or bilateral mastectomy of histopathologically confirmed mammary adenocarcinomas in 9 veterinary hospitals in USA, Canada and Italy between 1991 and 2014.
Sample Size	105 cats.
Intervention Details	Group 1 (n = 52): surgical treatment alone: 1a (n = 32): unilateral mastectomy 1b (n = 20): bilateral mastectomy (staged or single session). Group 2 (n = 53): surgical treatment and adjuvant chemotherapy: 2a (n = 28): unilateral mastectomy 2b (n = 25): bilateral mastectomy (staged or single session). Chemotherapy protocols: Doxorubicin (n = 44): Doxorubicin alone (n = 24) Doxorubicin + cyclophosphamide (n = 8) Doxorubicin + carboplatin (n = 12) Carboplatin alone (n = 3) Experimental liposomes (n = 2) Epirubicin (n = 2) Mitoxantrone (n = 1) Toceranib and chlorambucil (n = 1) Doxorubicin doses stated as “recommended doses” for 1–6 cycles (median 4 cycles). Doses and number of cycles for cyclophosphamide, carboplatin, experimental liposomes, epirubicin, mitoxantrone, toceranib and chlorambucil not stated.
Study design	Multicentre retrospective cohort study.
Outcome studied	Progression free survival time: time from mastectomy until documentation of local tumour progression, regional or distant metastasis, or tumour related death. Disease-specific survival time: time from mastectomy to tumour-related death. Tumour related death is defined as death or euthanasia as a result of local tumour recurrence, de novo tumour development, regional or distant metastasis, treatment or unknown causes. Post-operative complications.
Main findings (relevant to PICO question)	Treatment with adjuvant chemotherapy was not significantly associated with progression free survival time in a univariable analysis (p-value not included). Treatment with adjuvant chemotherapy was significantly associated with an increase in disease specific survival time in a univariable analysis (P < 0.25). Treatment with adjuvant chemotherapy was considered protective using a Cox multivariate analysis with death used as the predictor or final outcome (Hazard rate ratio (HR) 0.36; 95% CI 0.19–0.70). In this model, therefore, the use of adjuvant chemotherapy was associated with an increase in disease-specific survival time. A p-value was not included for this result and therefore its significance is unknown. Type of mastectomy, lymph node metastasis at the time of surgery, treatment with adjuvant chemotherapy and development of regional or distant metastasis were examined in the multivariate analysis.
Limitations	Retrospective design weak for clinical question. No placebo group. Population of cats in USA, Canada, and Italy may not be representative of population of cats in the UK. Seven centres were veterinary university hospitals and two were private and surgically specialised veterinary hospitals. Applicability to first opinion may be limited. Surgical technique and chemotherapy protocols were not standardised or repeatable. Data was not analysed per type of chemotherapy agent as they had very small populations of each. All chemotherapy agents were treated as one population and therefore it is unclear which chemotherapy protocol was protective. There is no information on which stage cats that were treated with adjuvant chemotherapy were in. This information may not be relevant as despite lymph node and/or distant metastasis adjuvant chemotherapy was still considered protective in the multivariate analysis. Grades based on histopathology were not included because the nomenclature used in the reports was not uniform. It is unknown if tumour grade had an effect on survival times. Evaluation of disease progression and tumour-related death was based on owner phone calls and the retrospective interpretation of clinical notes. The p-value for the multivariate Cox regression is not included and therefore its significance cannot be evaluated.

Table note...

**Ito et al. (1996):** 

Population	Cats diagnosed with malignant mammary tumours from 1982–1993 in the Veterinary Medical Centre of the University of Tokyo.
Sample Size	32 cats. The original sample size was 53 cats but only 34 had surgery +/- chemotherapy. Two cats from the surgery alone group were excluded because they were given chemotherapy.
Intervention Details	Group 1 (n = 16): cats with Stage I–III mammary carcinomas that had surgery alone (lumpectomy with involved mammary glands and/or adjacent mammary glands removed, unilateral or bilateral mastectomy). Group 2 (n = 16): Surgery + adjuvant chemotherapy: (n=10) Cyclophosphamide (1–2 mg/kg orally once a day for more than 5 weeks (5–18 weeks)). (n=5) Vincristine (0.5 mg/kg once a week for more than 5 cycles (6–10 cycles)). (n=1) Cyclophosphamide (1–2 mg-kg PO once daily for 12 weeks) and vincristine (0.5 mg/kg once a week for 8 cycles).
Study design	Retrospective cohort study.
Outcome studied	Survival time (time from diagnosis until the time of death by any cause or the date on which the cat was last known to be alive). Remission length (time from surgery to the time of detection of local recurrence or metastasis).
Main findings (relevant to PICO question)	Median rate of remission after surgery: Group 1: 12 months Group 2: 6 months. Median survival time: Group 1: 14 months Group 2: 10 months. The differences in rate of remission after surgery and median survival time found between groups were not statistically significant (P = 0.3997 and P = 0.2758, respectively).
Limitations	Retrospective design weak for clinical question. No placebo group. No power calculations were performed. Limited information on population (other comorbidities, current medications, etc.) Population of cats in Japan may not be representative of population of cats in the UK. Overrepresentation of Siamese cats in this study (34% or 18/53). Treatment options were not randomised or standardised. Inclusion of cats in different stages of cancer increases variability of results. Treatment performed in a referral hospital. Applicability to first opinion may be limited. Determining cause of death was based on patient’s clinical notes and owner phone calls. Follow up and clinical data was not always standardised or accessible and therefore it is difficult to determine if cause of death was due to progression of the disease. No information on what type of surgery cats that received chemotherapy had. Multivariate statistical analysis not performed.

Table note...

**McNeill et al. (2009):** 

Population	Cats with naturally occurring, biopsy confirmed feline mammary carcinomas diagnosed in 6 veterinary referral institutions and 3 general practices with specialty interest in feline medicine and surgery between 1989–2006 in the USA.
Sample Size	73 cats.
Intervention Details	Group 1 (n = 37): cats treated with surgery alone (local (n=24) vs radical mastectomy (n=13)). Group 2 (n = 36): cats treated with surgery and adjuvant doxorubicin-based chemotherapy (n = 4) with or without cyclophosphamide (n = 32). Doses and cycles were variable.
Study design	Multicentre retrospective cohort study.
Outcome studied	Disease free survival time (the time interval between the date of diagnosis and the first documentation of disease progression (either metastatic disease or local recurrence)). Overall survival time (time interval between date of diagnosis and date of death by any cause).
Main findings (relevant to PICO question)	Median disease-free survival: Group 1: 372 days Group 2: 672 days. Median survival time: Group 1: 1406 days Group 2: 848 days. The differences in median disease-free survival and median survival times found between groups were not statistically significant (P = 0.15 and P = 0.78, respectively). However, when comparing the type of surgery performed, within the subgroup of cats that underwent a radical unilateral mastectomy, those that had surgery plus adjuvant chemotherapy (n = 8) had significantly longer survival times than those that only had surgery (n = 10) (P = 0.03).
Limitations	Retrospective design weak for clinical question. No placebo group. No power calculations were performed. Limited information on population (other comorbidities, current medications, etc.). Population of cats in USA may not be representative of population of cats in the UK. Surgeries were performed in referral level hospitals and general practices with specialty interest in feline surgery. Applicability to first opinion may be limited. Treatment options were not randomised. The decision to start on adjuvant chemotherapy was left to owners. Study is not repeatable: Type of surgery, chemotherapy protocol and follow-up were not standardised. Not all animals were staged before treatment. If lymph nodes were palpably normal they were not sampled and these cats were classed as stage I. Microscopic metastases could have been missed. Some cats received rescue surgery and/or chemotherapy after relapse. Determining cause of death was based on patient’s clinical notes. Follow-up and clinical data was not always standardised or accessible. Only 3 cats had post-mortems to confirm cause of death as progression of disease. Inclusion of cats in different stages (I–III) increases variability of results. Multivariate statistical analysis was not performed.

Table note...

**Petrucci et al. (2021):** 

Population	Female cats with histopathologically confirmed mammary carcinomas that had complete tumour staging and radical mastectomy (unilateral or bilateral) between 2008–2018 in 8 veterinary teaching and private hospitals in Portugal.
Sample Size	137 cats.
Intervention Details	Group 1 (n = 80): cats treated with radical (unilateral or bilateral) mastectomy only. Group 2 (n = 34): cats treated with surgery and doxorubicin (1 mg/kg intravenously every 3 weeks for 5 treatment cycles). Group 3 (n = 23): cats treated with surgery, low dose metronomic oral cyclophosphamide (15 mg/m^2^ once daily) and meloxicam (0.05 mg/kg once daily) for 6 months.
Study design	Multicentre retrospective cohort study.
Outcome studied	Disease free interval (time from mastectomy until documentation of tumour progression). Overall survival (time from mastectomy until death by any cause). Toxicity and side effects of treatment.
Main findings (relevant to PICO question)	Median disease free interval: Group 1: 270 days Group 2: 226 days Group 3: 372 days. Median survival time: Group 1: 338 days Group 2: 421 days Group 3: 430 days. The difference in overall survival time (OST) and disease free interval (DFI) found between groups were not statistically significant (DFI: P = 0.280, OST: P = 0.186).
Limitations	Retrospective design weak for clinical question. No placebo group. No power calculations were performed. Disparity in cohort size: 58% (80/137) of cats only had surgical treatment while 24% (34/137) and 16% (23/137) of cats were treated with doxorubicin (group 2); and cyclophosphamide + meloxicam (group 3), respectively. Type of surgery, surgical technique, and chemotherapy protocols were not standardised or repeatable. Limited information on population (other comorbidities, current medications, etc.). Study population of cats in Portugal may not be representative of population of cats in the UK. Treatment options were not The decision to start on adjuvant chemotherapy was left to owners. Inclusion of cats in different stages (I–III) increased the variability of results. Overall survival was determined by death by any cause and therefore it is unclear if cause of death was always due to progression of disease.

Table note...

## Appraisal, application and reflection

Mammary neoplasms are the third most frequent tumour in cats and mainly affect female cats between the ages of 10–12 years (De Campos et al., 2014). Approximately 80–90% of feline mammary neoplasms are malignant carcinomas and are characterised by local invasion of surrounding tissues and a high rate of metastasis to local lymph nodes, lungs, and other sites (McNeill et al., 2009). Feline mammary carcinomas (FMCs) are classified into four stages (I–IV) based on the modified World Health Organization (WHO) clinical staging system. Higher stages have a poorer prognosis and shorter survival times (Petrucci et al., 2021; Gemignani et al., 2018, and Ito et al., 1996). FMCs are classified based on primary tumour size, lymph node involvement and presence of distant metastasis (McNeill et al., 2009). A histological grade (I–III) can also be used to further classify these neoplasms (De Campos et al., 2014). The current treatment of choice is radical bilateral or unilateral mastectomy. However, despite aggressive surgical treatment median survival times (ST) after surgery in stage III–IV FMCs is less than one year (Lo et al., 2019). Despite the poor prognosis of high-grade FMCs with radical surgery alone, there are only a small number of studies that explore the clinical benefits of using adjuvant chemotherapy.

The literature search for the PICO question revealed five relevant articles, all of which were observational and retrospective. Gemignani et al. (2018) is a multicentre study that includes data from referral university hospitals and private surgically specialised hospitals. The data for De Campos et al. (2014) and Ito et al. (1996) studies were obtained from one referral teaching hospital in Brazil and Japan, respectively. Petrucci et al. (2021) and McNeill et al. (2009) were the only studies that included data from referral teaching hospitals and private veterinary hospitals. Crucially, the results obtained may not be applicable to first opinion clinical practice given that most of the studies were performed in referral or specialist level hospitals. Surgical technique and surgeon experience could affect surgical outcome and survival times. In this instance, chemotherapy may play a bigger role with less experienced surgeons in first opinion practices where complete surgical excision may not be achieved. Interestingly, however, in McNeill et al. (2009) study 59% (43/73) of surgeries were performed in general practice and there were no statistically significant differences found between treatment groups in regard to the type of hospital where surgery was performed. It is also worth noting that none of the studies were performed in the United Kingdom and therefore the results may not be applicable to a population of British cats. The prevalence of different cat breeds within a feline population could have an effect on the outcomes studied. Hayes et al. (1981) found that Siamese cats had twice the risk of developing mammary carcinomas compared to other breeds. This breed was overrepresented in Ito et al. (1996) cohort group and could have had an effect on this study’s overall survival times.

All five papers appraised use the modified WHO staging system in order to stage their cohort groups. However, given the retrospective nature of all of the papers appraised staging was not standardised. In Petrucci et al. (2021) study all cats had pre-surgical thoracic radiographs and an abdominal ultrasound or a full body Computed Tomography (CT). In McNeill et al. (2009) paper 92% (67/73) of cats had pre-operative thoracic radiographs but only 32% (23/73) had an abdominal ultrasound. Moreover, if local lymph nodes were palpably normal or not removed during surgery these were deemed to be normal and therefore the cat was automatically classified as having a stage I FMC. Gemignani et al. (2018), De Campos et al. (2014), and Ito et al. (1996) make reference to the WHO staging system. However, there is no further information on how many cats had pre-surgical imaging or whether local lymph nodes were sampled for cytology. The lack of a standardised pre-operative staging system could result in local and distant metastases being missed and the incorrect classification of cats into lower FMC stages. This potentially underestimates the effect of adjuvant chemotherapy on low stage FMCs and could inadvertently include cats with stage IV FMCs in these studies (De Campos et al. (2014), Gemignani et al. (2018), and Ito et al. (1996)). In both instances this would result in a reduction in STs in all cohort groups. Conversely, Matos et al. (2012) further argue that some nodules that are not sampled and assumed local or distant metastasis could actually be benign or even different neoplasms. Therefore, any future prospective studies on FMCs need to have a standardised pre-surgical staging protocol (including sampling of local lymph nodes and suspected distant metastasis). Unfortunately, a cohort group with these specifications is difficult to obtain in a retrospective and multicentre study.

Cats with stage I, II, and III FMCs were included in four of the papers appraised (Gemignani et al., 2018; Ito et al., 1996; McNeill et al., 2009; Petrucci et al., 2021), though most of the cats included in McNeill et al. (2009) were stage I–II. De Campos et al. (2014) is the only paper that exclusively considers cats with stage III FMCs. None of the papers include cats with known distant metastasis (stage IV). In those papers that include cats in different stages it is difficult to ascertain whether STs are influenced by severity of disease. Both Petrucci et al. (2021) and Gemignani et al. (2018) concluded that higher stages (stages III–IV) have lower STs compared to stages I–II. In Petrucci et al. (2021) multivariate analysis cats in stage III had 2.4 times higher risk of recurrence and 3.1 times higher risk of death compared to cats in stage I (adjusting for other variables). Therefore, stage is an important variable in determining STs and should be taken into account in any statistical analysis of FMCs. This is especially relevant in those studies where a multivariate analysis is not performed and STs are considered independently of variables such as lymph node metastasis at the time of surgery, development of regional or distant metastasis or type of mastectomy (McNeill et al., 2009 and Ito et al., 1996).

Any future prospective study should look at the effect of adjuvant chemotherapy on STs in populations of cats with the same tumour stage. Crucially, this would give us a better understanding as to whether the use of adjuvant chemotherapy is most useful in cats with advanced or low-stage FMCs. McNeill et al. (2009) suggest that cats with stage I or II FMCs already have a good prognosis with radical surgery alone so using adjuvant chemotherapy may be less justified, especially given the potential side-affects as well as the economic and emotional commitment required from owners associated to this additional therapy. McNeill et al. (2009) therefore argue that adjuvant chemotherapy plays a bigger role in prolonging ST in stage III FMCs. Alternatively, Borrego et al. (2009) argue that staging has its limitations and one cannot underestimate microscopic metastasis that may go undiagnosed during initial staging. In their study they found that some cats with stage I-II FMCs that had complete margins post-operatively later developed local and/or distant metastasis. This suggests that a multimodal treatment protocol including radical surgery and adjuvant chemotherapy should be implemented in all cats with FMCs, regardless of stage, in order to achieve maximum STs. However, Borrego et al. (2009) study is also retrospective and therefore lacks standardisation of pre-surgical staging. The prognostic significance of micrometastasis in FMCs, therefore, remains uncertain.

Type of surgery has also been shown to be significantly associated to STs in FMCs, with radical mastectomies resulting in prolonged disease-free intervals compared to lumpectomies (Gemignani et al., 2018, Petrucci et al., 2021). Petrucci et al. (2021) and Gemignani et al. (2018) only include cats that have had radical unilateral or bilateral mastectomies. De Campos et al. (2014), McNeill et al. (2009), and Ito et al. (1996), however, include cats that have had local as well as radical unilateral or bilateral mastectomies. In those papers that include cats that had both local and radical surgery STs could vary as a consequence of the type of surgery performed. Interestingly, McNeill et al. (2009) found that in those cats treated with unilateral mastectomies STs were significantly longer in the surgery plus adjuvant chemotherapy group. This statistical significance was not found when type of surgery was not taken into account. This is a surprising result given that Gemignani et al. (2018) argue that bilateral radical mastectomies are protective compared to unilateral mastectomies. However, one should interpret McNeill et al. (2009) results cautiously given that the population of cats that had unilateral mastectomies (n = 18/73) was very small and likely underpowered. Moreover, due to the fact that a multivariate statistical analysis was not performed it is difficult to evaluate the effect that other variables (such as surgeon experience, lymph node metastasis, or histologic grade) had on STs in this group. None of the papers that include lumpectomies in their surgical protocol perform a multivariate analysis, so it is impossible to ascertain whether type of surgery had an effect on STs in these studies (De Campos et al. (2014), Ito et al. (1996) and McNeill et al. (2009)).

The chemotherapy protocols in all studies were also clinician dependent and therefore very variable. For example, in Petrucci et al. (2021) paper not all cats in group 2 received the same number of chemotherapy cycles, while the cats in McNeill et al. (2009) study received variable doses and number of cycles as part of their chemotherapy protocol. Furthermore, the chemotherapy agents, doses and/or number of cycles vary between papers and therefore comparing results between studies is impossible. The chemotherapeutic agents used range from doxorubicin, cyclophosphamide, carboplatin, and vincristine but the predominant chemotherapeutic agent in most papers is doxorubicin (Gemignani et al., 2018; McNeill et al., 2009 and Petrucci et al., 2021). However, the dose and number of doxorubicin cycles used vary between papers and within the same studies. In McNeill et al. study (2009) 81% (29/36) of cats in the group received more than or equal to 3 cycles of treatment, while 67% (24/36) received 4 or more cycles. Novosad et al. (2006) and Borrego et al. (2009) argue that the number of doxorubicin cycles used is significantly associated with disease free intervals (DFI). They argue that cats that received fewer than 4 cycles of doxorubicin had a statistically lower DFI than those cats that received 4 or 5 cycles. Therefore, the STs in McNeill et al. (2009) could potentially be longer if all cats had received an adequate number of doxorubicin cycles. This may be complicated in practice given that owners and/or veterinarians may elect to discontinue chemotherapy treatment if considerable side-effects are noted or due to monetary constraints.

In addition, there is also a strong bias in all the studies appraised, given that the decision to perform adjuvant chemotherapy is left to owners and clinicians. One could argue that the owners that elect more aggressive treatment (i.e. chemotherapy) are more likely to perform more frequent monitoring (and detect progression of disease and other comorbidities sooner) and less likely to elect cessation of treatment which could result in prolongation of STs. Conversely, clinicians may elect to use chemotherapy in those cases with more advanced FMC stages where surgical treatment does not achieve a long enough ST on its own. Therefore, selecting for cases that have an intrinsically poorer ST and consequently reducing the STs in the adjuvant chemotherapy groups. It is important that future prospective studies are double-blinded and randomised in order to minimise owner and clinician bias.

All the papers appraised use median survival time as a studied outcome. Gemignani et al. (2018), Ito et al. (1996), McNeill et al. (2009) and Petrucci et al. (2021) further include DFI (also described as disease free survival, remission length and progression free interval) as a studied outcome. In a population of cats with cancer DFI is a more useful outcome to study. Using survival times as a measurable outcome has multiple limitations. Firstly, STs carry a strong owner bias as it is dependent on owners electing to stop treatment or euthanise based on perception of reduced quality of life, side-effects of treatment or monetary constraints. Ito et al. (1996), McNeill et al. (2009) and Petrucci et al. (2021) study overall survival times with death by any cause. This is not an adequate measurable outcome for the clinical question at hand given that many of the deaths documented in their studies could be due to progression of other comorbidities. This is especially relevant in the cohort groups for all of the studies appraised given that most cats were geriatric at the time of diagnosis. In De Campos et al. (2014) and Gemignani et al. (2018) disease specific survival times are measured. However, given the retrospective nature of these studies it is also difficult to unequivocally determine the cause of death of the cats. Most of the studies rely on clinical notes and/or owner phone calls in order to determine cause of death. None of the studies include an owner questionnaire template and therefore one cannot assess if the questions asked were biased. In any future perspective studies on FMCs, sampling tissue that causes suspicion for local or distant metastases and performing post-mortems with histopathology on all cats may be crucial to determining disease progression and cause of death.

Furthermore, measuring DFI does not come without limitations as it is very much influenced by the ability of disease progression to be noticed by owners and/or clinicians. Some owners may be more aware than others of the clinical signs (i.e. coughing, exercise intolerance) that should prompt a veterinary visit, while others may be more comfortable in periodically palpating their cats’ mammary glands in order to monitor for progression. Owner compliance, available funds, and owner availability may strongly influence how regularly a patient is seen by a veterinary surgeon. Moreover, disease progression is not confirmed using cytology or histopathology in all of the cats in the studies appraised. In Petrucci et al. (2021) only 40.3% (29/72) of local or distant metastasis are confirmed with cytology, 30.5% (22/72) had confirmed metastasis by histological evaluation post-mortem and 29.2% (21/72) of metastasis were assumed using imaging alone. Finally, in Petrucci et al. (2021)s, cats receiving adjuvant chemotherapy were seen every 3 weeks as opposed to cats that only had surgery who were seen at least every 3 months. More regular veterinary visits in the chemotherapy groups could increase detection of disease progression. This could decrease DFIs and potentially also reduce STs given that some owners may elect to euthanise sooner if there is evidence of disease progression despite use of chemotherapy.

The literature search for the PICO question showed that there are very few studies on the use of adjuvant chemotherapy in FMCs and none of them offer enough strength of evidence to conclude whether it makes a difference to survival times. Only Gemignani et al. (2018) found that the use of chemotherapy had a statistically significant effect on prolonging disease specific ST and therefore was considered protective in the multivariate analysis. However, similar to the other studies appraised, it does not have enough strength of evidence to suggest that cats with mammary carcinomas that have undergone surgical treatment will have a longer survival time if treated with adjuvant chemotherapy. Larger prospective, double-blinded and randomised studies are needed to properly evaluate the clinical relevance of adjuvant chemotherapy in feline mammary carcinomas.

## Methodology

**Search strategy:** 

Databases searched and dates covered	Medline on OVID 1946 to April 2024 CAB abstracts on OVID 1973 to April 2024
Search terms	Medline Search: Cats/ (cat or cats or feline*).tw. 1 or 2 Mammary Neoplasms, Animal/ (mammary adj3 (carcinoma* or cancer* or neoplasm* or tumor* or tumour*)).tw. 4 or 5 3 and 6 exp Mastectomy/ (mastectomy or mastectomies or mammectomy or mammectomies or remov* or surger*).tw. 8 or 9 7 and 10 exp Drug Therapy/ (chemotherap* or drug therap* or pharmacotherap*).tw. 12 or 13 11 and 14 CAB Abstracts: exp cats/ (cat or cats or feline*).tw. 1 or 2 mammary gland neoplasms/ (mammary adj3 (carcinoma* or cancer* or neoplasm* or tumor* or tumour*)).tw. 4 or 5 3 and 6 mastectomy/ (mastectomy or mastectomies or mammectomy or mammectomies or remov* or surger*).tw. 8 or 9 7 and 10 exp drug therapy/ (chemotherap* or drug therap* or pharmacotherap*).tw. 12 or 13 11 and 14 limit 15 to english language
Dates searches performed	12 Apr 2024

Table note placeholder

**Exclusion / inclusion criteria:** Caption placeholder

	Exclusion / Inclusion criteria
Exclusion	Case report/letter to the editor/book chapter, not relevant to PICO question.
Inclusion	Relevant to PICO question, study population divided into those that had surgery alone versus surgery plus adjuvant chemotherapy.

Table note placeholder

**Search Outcome:** Caption placeholder

**Data**base	Number of results	Excluded – case report/letter to the editor/book chapter	Excluded – not relevant to the PICO question	Total relevant papers
Medline OVID	25	0	20	5
CAB Abstracts	51	34	14	3
Total relevant papers when duplicates removed				5

Table note placeholder

## ORCiD

Gabriela Gonzalez-Ormerod: https://orcid.org/0000-0001-5043-1069

## Conflict of interest

The author declares no conflicts of interest.

## Disclaimer

Knowledge Summaries are a peer-reviewed article type which aims to answer a clinical question based on the best available current evidence. It does not override the responsibility of the practitioner. Informed decisions should be made by considering such factors as individual clinical expertise and judgement along with patient’s circumstances and owners’ values. Knowledge Summaries are a resource to help inform and any opinions expressed within the Knowledge Summaries are the author’s own and do not necessarily reflect the view of the RCVS Knowledge. Authors are responsible for the accuracy of the content. While the Editor and Publisher believe that all content herein are in accord with current recommendations and practice at the time of publication, they accept no legal responsibility for any errors or omissions, and make no warranty, express or implied, with respect to material contained within. For further information please refer to our Terms of Use.
